# Recent Advances in the Treatment of Genetic Forms of Parkinson’s Disease: Hype or Hope?

**DOI:** 10.3390/cells12050764

**Published:** 2023-02-27

**Authors:** Francesco Cavallieri, Rubens G. Cury, Thiago Guimarães, Valentina Fioravanti, Sara Grisanti, Jessica Rossi, Edoardo Monfrini, Marialuisa Zedde, Alessio Di Fonzo, Franco Valzania, Elena Moro

**Affiliations:** 1 Neurology Unit, Neuromotor & Rehabilitation Department, Azienda USL-IRCCS of Reggio Emilia, 42123 Reggio Emilia, Italy; 2 Department of Neurology, School of Medicine, University of São Paulo, São Paulo 05403-000, Brazil; 3 Clinical and Experimental Medicine PhD Program, University of Modena and Reggio Emilia, 41125 Modena, Italy; 4 Neurology Unit, Fondazione IRCCS Ca’ Granda Ospedale Maggiore Policlinico, 20122 Milan, Italy; 5 Division of Neurology, CHU of Grenoble, Grenoble Institute of Neurosciences, Grenoble Alpes University, 38700 Grenoble, France

**Keywords:** DJ1, genetic, GBA, LRRK2, Parkinson’s disease, PINK1, PRKN, SNCA, treatment

## Abstract

Parkinson’s disease (PD) is a multifarious neurodegenerative disease. Its pathology is characterized by a prominent early death of dopaminergic neurons in the pars compacta of the substantia nigra and the presence of Lewy bodies with aggregated α-synuclein. Although the α-synuclein pathological aggregation and propagation, induced by several factors, is considered one of the most relevant hypotheses, PD pathogenesis is still a matter of debate. Indeed, environmental factors and genetic predisposition play an important role in PD. Mutations associated with a high risk for PD, usually called monogenic PD, underlie 5% to 10% of all PD cases. However, this percentage tends to increase over time because of the continuous identification of new genes associated with PD. The identification of genetic variants that can cause or increase the risk of PD has also given researchers the possibility to explore new personalized therapies. In this narrative review, we discuss the recent advances in the treatment of genetic forms of PD, focusing on different pathophysiologic aspects and ongoing clinical trials.

## 1. Introduction

Parkinson’s disease (PD) is a complex and manifold neurodegenerative disease. Its pathology is characterized by a prominent early death of dopaminergic neurons in the pars compacta of the substantia nigra (SNpc) and the presence of Lewy bodies containing aggregated α-synuclein encoded by the SNCA gene [1,2]. Currently, the diagnosis of PD is clinical and based on the presence of bradykinesia, eventually being associated with rigidity and resting tremor [1]. It is well known that the neurodegenerative process of PD starts several years before the onset of motor symptoms [3]. This prodromal phase is heterogeneous and depends on the phenotype of PD (according to the “brain first, body first” onset of pathology) [4]. The PD clinical course and the response to treatment vary just like its etiology, which is multifactorial and complex [5]. It is currently hypothesized that pathogenic mechanisms linked with sex, genomic, epigenetic, and environmental factors lead to several alterations at the cellular level [6,7,8,9]. These may include: conformational changes with malfunction and accumulation of key proteins due to abnormalities in their clearance systems (ubiquitin–proteasome system; lysosome- and chaperone-mediated autophagy); dysregulation of mitochondrial function and oxidative stress; loss of trophic factors; alterations of intracellular Ca^2+^ homeostasis; and finally neuroinflammation [2,9,10]. Nevertheless, the pathological aggregation and spread of α-synuclein is considered the key event in PD pathogenesis [11,12]. This protein, mainly expressed in the brain, is fundamental for neurotransmitter release and synaptic vesicle function [13]. It has been hypothesized that environmental factors (viruses, bacteria, toxins, etc.) might start α-synuclein’s pathological accumulation, likely favored by a genetic predisposition [14,15,16]. The treatment of PD is currently only symptomatic, mainly focused on an improvement in motor and non-motor signs and symptoms [17]. However, PD management requires a multidisciplinary and holistic approach that should integrate pharmacological and non-pharmacological treatment. Among the latter, rehabilitative therapy and exercise should be implemented during all phases of PD [17]. Indeed, recent studies have shown the presence of a dose–response association between physical activity and all-cause mortality in PD patients. This underlies the need to increase and maintain physical activity in PD [18], and this can play a preventative and maintenance role regarding physical fitness and mental health [19].

**Figure 1:**
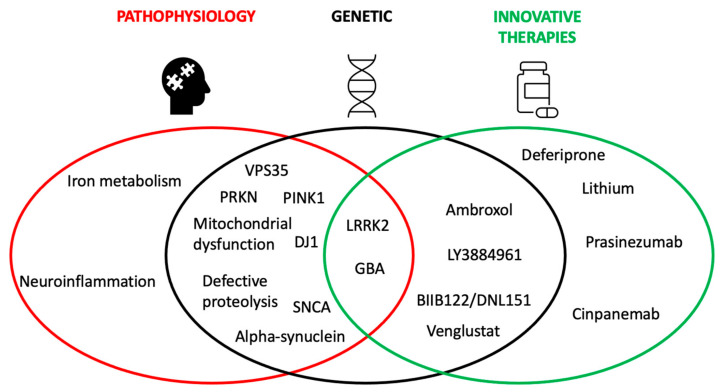
Interplay between genetic contributions to PD, pathophysiology, and innovative therapies. The importance of the genetic contribution to PD pathogenesis is twofold. From one side, the identification of genetic variants that can cause or increase the risk of PD can elucidate the different pathophysiological mechanisms involved in the disease. From another side, this knowledge can help better investigate potentially effective treatments and explore new personalized therapies. As an example, mutations in the GBA or LRRK2 genes have led to a better understanding of PD pathophysiology but also to the development of innovative experimental therapies (i.e., ambroxol, venglustat, BIIB122/DNL151, LY3884961). On the other hand, DJ1, PINK,1 and SNCA have allowed us to improve our knowledge about the underlying pathophysiology of PD but until now no experimental treatments specifically targeting these genes have been developed. Some other innovative therapies, such as prasinezumab, cinpanemab, deferiprone and lithium, are not focused on the genetic aspects of the disease but on the common pathophysiological mechanisms of PD. Abbreviations: glucocerebrosidase: GBA; leucine-rich repeat kinase 2: LRRK2; Parkin: PRKN; PTEN Induced Kinase-1: PINK1; VPS35: Vacuolar protein sorting ortholog 35.

Mutations associated with a high risk for PD (monogenic PD) underlie 5% to 10% of all PD cases [20,21,22]. However, this percentage tends to increase over time as a result of the continuous identification of new genes associated with PD [5,23,24]. Besides PD-related genes, the SNCA gene was the first gene associated with inherited PD [12]. In more recent years, mutations in the leucine-rich repeat kinase 2 (LRRK2) and parkin (PRKN) genes were found to be the most common causes of dominantly and recessively inherited forms of PD, respectively [20,21,22]. Heterozygous mutations in the β-glucocerebrosidase gene (GBA) currently represent the greatest genetic risk factor for developing PD [25]. An updated list of the genes associated with PD is provided in Table 1 [20]. The importance of the genetic contribution to PD pathogenesis is twofold, as illustrated in Figure 1. On the one hand, the identification of genetic variants linked to PD may elucidate the different pathophysiological mechanisms involved in the disease. On the other hand, this knowledge can help to investigate potential new experimental and personalized therapies tailored to the genetic profile of an individual patient [10]. In this narrative review, we discuss the recent advances in the treatment of the most relevant genetic forms of PD, focusing on different pathophysiologic aspects that have driven ongoing clinical trials.

## 2. GBA Gene 

### 2.1. Pathophysiological Mechanisms

The GBA gene is located on chromosome 1 (1q21) and encodes the lysosomal enzyme glucocerebrosidase (GCase), which is involved in the metabolism of glucosylceramide (GL-1), a basic glycolipid component of the cell membrane [25]. Biallelic mutations in the GBA gene have been classically associated with Gaucher’s disease (GD), a systemic disorder with a varying degree of central nervous system (CNS) involvement [25]. After observing an increased risk of PD in patients with GD [26,27], several large-scale genetic studies have also demonstrated that heterozygous variants in the GBA gene are the most important genetic risk factor for developing PD. Indeed, heterozygous GBA variants account for 5–30% of PD cases depending on the population and age [27,28,29,30]. To date, more than 300 GBA variants have been associated with PD, with an overall odds ratio (OR) for developing the disease of approximately 3.5–6 [27]. This OR seems to be directly linked to the severity of GBA mutations; indeed, severe GBA mutations (i.e., L444P, IVS2+1G>A, c.84dupG, V394L, D409H, RecTL, RecNCil) are associated with a higher risk of PD compared to mild ones (i.e., N370S) [25]. In addition, the severity of GBA mutations may also influence the clinical phenotype and the severity of the disease [27,28,29,31]. PD patients carrying severe mutations have an earlier age of onset and greater risk of dementia, impulsive–compulsive behavior (ICB), and delusions when compared to PD patients carrying mild mutations in the GBA gene [27,28,29,31]. The molecular mechanisms underlying the pathogenesis of GBA-PD are complex and not yet fully understood. The direct correlation between the severity of both the clinical course and the GBA variants (on the basis of the deleterious effect on GCase enzymatic activity) support the hypothesis of a key pathogenic role played by the loss of GCase function. However, the scenario is far from being so defined. PD patients harboring GBA variants such as E326K, which has a less-pronounced effect on GCase enzyme activity, do not appear to have a more benign clinical course [29,31]. Several reports describe an association between this variant and a higher risk of cognitive problems [29,31]. In addition, if the risk of PD depended on the extent of the residual GCase activity, most patients with GD would be expected to develop PD. However, that does not seem to happen [29,31]. The link between GBA mutations and PD still requires more effort in order to tackle the right molecular mechanism that is dysfunctional in the genesis of PD. The mutated GCase is not able to fold properly in the endoplasmic reticulum (ER), causing the protein to accumulate in this cellular compartment [25]. This leads to two main relevant consequences. Firstly, the accumulation of misfolded GCase protein in the ER may directly lead to ER oxidative stress with subsequent neuronal loss in dopaminergic neurons [32]. Secondly, the reduction in endolysosomal GCase activity may cause α-synuclein accumulation [25,32]. In addition, the accumulation of GL-1, due to low endolysosomal GCase activity, can also play a role, affecting the membrane fluidity of lysosomes and accelerating the formation of toxic α-synuclein oligomers, which in turn may block the ER–Golgi trafficking of GCase and lead to further GL-1 accumulation [33]. The failure of the endolysosomal and autophagic pathways is considered one the most important alterations at a cellular level in PD [2,10,25]. This is not surprising, since these scavenger systems are crucial for the degradation of α-synuclein, whose accumulation in the dopaminergic neurons is one of the hallmarks of PD [25].

### 2.2. Novel Therapeutic Approaches and Ongoing Clinical Trials

Novel therapeutic approaches in GBA-PD are based on both the attempt to increase GCase activity through gene therapy or GCase enhancers and to reduce substrate accumulation [34]. Table 2 summarizes the ongoing clinical trial in GBA-PD patients. 

The first novel therapeutic approach developed in GBA-PD patients was based on molecular chaperones, a class of protein that may facilitate the refolding of their substrates [35]. Chaperones may help to refold mutant GCase inside the ER, facilitating trafficking and increasing GCase levels in lysosomes [27,36]. Ambroxol is an inhibitory chaperone that mobilizes the sequestered mutant GCase from the ER, inducing a conformational change that facilitates transport to lysosomes and the recovery of GCase lysosomal function. In vitro and in vivo studies have confirmed that ambroxol can increase GCase activity and reduce α-synuclein levels [37]. In a phase 2, open-label study (NCT02941822) involving 17 PD patients with and without GBA mutations, ambroxol (at an escalating oral dose to 1.26 g per day) was able to both cross the blood–brain barrier (BBB) and to increase the GCase and α-synuclein concentrations in the cerebrospinal fluid (CSF) [38]. Based on these promising results, two phase 2 clinical trials are ongoing (NCT05287503, NCT02914366). In particular, NCT02914366 is a 52-week, randomized, placebo-controlled, quadruple-masking trial that is testing the hypothesis that oral ambroxol (1050 mg/daily) may improve cognitive and motor symptoms in patients with PD dementia (PDD). In addition, the multicenter, randomized, double-blind, placebo-controlled NCT05287503 trial (Ambitious study) is investigating whether the prolonged administration of high-dose oral ambroxol is able to change GCase activity and α-synuclein CNS levels and to reduce the progression of cognitive decline and motor disability in a cohort of 60 PD-GBA patients. Aside from ambroxol, other small-molecule chaperones (GC00188758, NCGC607, quetiapine, PGRN, HSP70, arimoclomol, LTI-291) have been investigated, but are so far supported by limited preclinical evidence [27].

Gene therapy in GBA-PD patients relies on the delivery of a normal GBA gene using an adenoassociated virus (AAV) vector [39]. The efficacy of this approach has been confirmed in different animal models of PD-GBA [39,40,41,42], showing a significant reduction in both α-synuclein accumulation and CNS inflammation [39]. Based on these premises, the first experimental gene therapy in PD-GBA patients has been recently developed [39]. This investigational drug (PR001) is composed of a viral vector (adenoassociated virus serotype 9) containing a codon-optimized plasmid encoding a wild-type human GBA gene, proved to be able to increase GCase activity, decrease glycolipid substrate accumulation, and improve motor abnormalities in GBA-PD models in vivo [27,39]. This has led to the development of the J3Z-MC-OJAA study, a Phase 1/2a, multicenter, open-label, ascending-dose, first in-human study that will evaluate the safety of intratecal LY3884961 administration in patients with moderate to severe PD with at least one pathogenic GBA mutation (NCT04127578). Two escalating dose cohorts (low-dose and high-dose) will be studied, and patients will be evaluated for the effect of LY3884961 on safety, tolerability, immunogenicity, biomarkers, and clinical efficacy measures with a 5-year follow-up. 

Another therapeutic approach in GBA-PD is focused on the reduction of GL-1 accumulation [25]. Venglustat is a potent, CNS-penetrant inhibitor of glucosylceramide synthase that can reduce the formation of GL-1 [33]. The efficacy of venglustat in GBA-PD patients was explored in the MOVES-PD trial, a randomized, double-blinded, placebo-controlled, dose-escalation study (NCT02906020). The first part of the study included 29 GBA-PD patients, and showed that venglustat had a favorable safety and tolerability profile [33]. However, the second part of the MOVES-PD trial (NCT02906020), which included 273 early-PD patients, did not meet the study’s primary objective, i.e., the efficacy on motor symptoms (primary endpoints: MDS-UPDRS part II–III). In particular, a progressive deterioration in clinical outcomes was noticed over time in the treatment arm compared to the placebo, leading to a premature interruption of the study [27].

## 3. LRRK2 Gene

### 3.1. Pathophysiological Mechanisms

Pathogenic mutations in the LRRK2 gene are common genetic risk factors for both familial and sporadic adult-onset PD [43]. Up until now, over 50 different LRRK2 variants have been identified [44]. The most common mutation, LRRK2-G2019S, accounts for up to 6–40% of familial PD cases depending on the ethnic group, and up to 2% of all sporadic cases [43]. PD patients with the G2019S mutation usually present with a substantial clinical overlap with idiopathic PD and a similar rate of progression [5]. However, they may more commonly have a postural-instability/gait-difficulties phenotype, levodopa-induced dyskinesias, and fewer nonmotor manifestations [5]. LRRK2 is located at chromosome 12q12 and encodes a multidomain protein of 2527 amino acids harboring different catalytic domains, including the MAPKKK kinase domain, the Ras of complex (ROC) GTPase domain, and the C-terminal of ROC (COR) domain [44]. Seven missense LRRK2 mutations have been identified as pathogenic, including R1441G, R1441C, R1441H, Y1699C G2019S, R1628P, G2385R, and I2020T, which are mainly located in the catalytic domains of the LRRK2 gene [45]. LRRK2 is widely expressed in several tissues including the brain, lungs, heart, and kidney [45]. At the brain level, LRRK2 mRNA and proteins are highly expressed in dopamine-innervated areas including the cerebral cortex, striatum, cerebellum, and hippocampus, while at low levels in dopaminergic neurons of the substantia nigra and ventral tegmental area [45]. At the cellular level, the LRRK2 protein is mainly found throughout the cytoplasm associated with various intracellular membranes and vesicular structures (i.e., early endosomes, lysosomes, plasma membrane and synaptic vesicles, ER, Golgi complex, and outer mitochondrial membrane) [45]. 

Through the phosphorylation of several substrates, LRRK2 is involved in several cellular functions including late-stage endocytosis, lysosomal trafficking, cytoskeletal remodeling, and synaptic-vesicle endocytosis [43,46]. The current understanding of LRRK2 functions and pathogenicity in PD is still incomplete [5]. It is believed that increased LRRK2 activity may raise the risk of PD because the increased kinase activity has been associated with nigrostriatal degeneration and Lewy body (LB) formation [5,43,44,45]. In addition, the G2019S mutation, located in the kinase domain of the gene, has been associated with increased phosphorylation activity in vivo [5]. In primary neuronal cultures, overexpression of LRRK2 G2019S, I2020T, R1441C, or Y1699C consistently induces neuronal toxicity, as evidenced by neurite shortening, cell death, and impaired functions of intracellular organelles [43]. Furthermore, many of these phenotypes may be alleviated by introducing kinase-inactive or guanosine triphosphate (GTP)-binding-deficient mutations and/or treatment with chemical inhibitors of LRRK2 [43]. LRRK2 may also cause α-synuclein neurotoxicity by increasing its propagation and aggregation in a kinase-dependent manner with the contemporary reduction in its clearance [47]. LRRK2 may also play a role in cell-to-cell transmission and long distance spreading of α-synuclein, presumably through regulation of the release, uptake, and lysosomal/proteasomal degradation of the protein [43]. LRRK2 can also interact with several other proteins throughout the endolysosomal pathway, and its excessive expression levels or kinase activity may disrupt vesicle trafficking and protein degradation [48].

### 3.2. Novel Therapeutic Approaches and Ongoing Clinical Trials

The objective of the novel therapeutic approaches in LRRK2-PD is to reduce the pathological excessive kinase activity of the mutated gene. Table 3 summarizes the ongoing clinical trial in LRRK2-PD. The CNS-penetrant, selective, small-molecule LRRK2 kinase inhibitor DNL201 was able to inhibit LRRK2 kinase activity and improve lysosomal function in preclinical models [49]. In one phase 1 and one phase 1b clinical trial, which included 122 healthy volunteers and 28 PD patients, DNL201 (at single and multiple doses) inhibited LRRK2, was well tolerated, and showed robust CSF penetrance [49]. The safety, tolerability, pharmacokinetics, and pharmacodynamics of multiple oral doses of LRRK2 kinase inhibitor BIIB122/DNL151 were assessed in PD patients in a phase 1 study (NCT04056689) whose results are still pending. This molecule is now under investigation in two other ongoing trials. The objective of the phase 2b, multicenter, randomized, double-blind, placebo-controlled study LUMA (NCT05348785) is to assess the safety of BIIB122 oral tablets (225 mg once daily) and the possible impact of the drug on disease progression. A total of 640 early-stage PD patients without mutations in the LRRK2 gene will be enrolled. In addition, the phase 3, multicenter, randomized, double-blind, placebo-controlled study LIGHTHOUSE (NCT05418673) will aim to determine the safety profile and the efficacy of BIIB122/DNL151 to slow the progression of disease in 400 LRRK2-PD patients. The use of antisense oligonucleotides (ASOs) for LRRK2 inhibition represents another therapeutic approach under investigation in LRRK2-PD patients. This approach is supported by preclinical studies that showed that the administration of LRRK2 ASOs to the brain was able to reduce LRRK2 protein levels and fibril-induced α-synuclein inclusions [50]. Furthermore, mice exposed to α-synuclein fibrils treated with LRRK2 ASOs showed more tyrosine hydroxylase (TH)-positive neurons compared to control mice, suggesting that LRRK2 ASOs treatment could be a potential therapeutic strategy for preventing PD-associated pathology [50]. Based on these premises, the ongoing phase 1 single- and multiple-ascending-dose study REASON (NCT03976349) will assess the safety, tolerability, and pharmacokinetic profile of intrathecal injections of the LRRK2 ASO inhibitor BIIB094 in PD patients with and without LRRK2 mutations. 

## 4. SNCA Gene

### 4.1. Pathophysiological Mechanisms

Generally, patients carrying SNCA mutations present with early-onset parkinsonism with severe and early non-motor symptoms, including cognitive decline. However, many different PD phenotypes have been related to SNCA mutations [51,52]. Indeed, while in whole-gene multiplications, the number of SNCA copies clearly correlates with the disease severity, supporting the notion of a “dosage effect”, missense mutations cause more complex phenotypes with mutation-specific trends in clinical presentations [52]. α-synuclein, encoded by the *SNCA* gene, is a 14-kDa protein involved in synaptic vesicle release [11], mitochondrial function, and intracellular trafficking and is a potential chaperone [53]. The deposition of α-synuclein oligomers and fibrils disrupts synaptic-vesicle trafficking at the presynaptic terminal leading to an impairment of dopamine release [54] and dopamine transporter (DAT) function [54]. The deposition of α-synuclein oligomers may also impair mitochondrial function, leading to increased mitophagy and mitochondrial DNA damage and decreasing the mitochondrial biogenesis factor peroxisome proliferator-activated receptor γ co-activator 1α (PGC-1α) [55]. This is supported by animal models carrying A53T and A30P α-synuclein mutations [56,57]. A-synuclein deposition can also disrupt ER and Golgi trafficking, with a subsequent reduction in lysosomal enzyme levels, which in turn impairs the autophagic degradation of damaged organelles and protein aggregates [11]. Finally, α-synuclein fibrils activates microglia via TLR2 (Toll-Like Receptor 2), resulting in the activation of NF-kB and MAPK, and the production and release of pro-inflammatory mediators. 

### 4.2. Novel Therapeutic Approaches and Ongoing Clinical Trials

Table 4 summarizes the ongoing clinical trials focused on α-synuclein. The treatment strategies may be subdivided into different approaches. One strategy relies on the reduction of α-synuclein synthesis using small interfering RNAs (siRNA) that target α-synuclein mRNA or antisense oligonucleotides (ASO); the latter has shown promising results in animal models [58,59]. However, no related clinical trial is currently ongoing. Another approach is based on the increase in the degradation of α-synuclein aggregates through autophagy and lysosomal function. As an example, the overexpression of lysosomal transcription factor EB (TFEB) in rats expressing α-synuclein decreases its oligomer’s levels, preventing organelle dysfunction and neurodegeneration [60]. This approach includes other possible targets including the mammalian target of rapamycin (mTOR) signalling and the cellular homolog of ABL1 (c-Abl) [10]. Indeed, α-synuclein overexpression inhibits autophagy by increasing mTOR activity, which can be modulated or inhibited by different substances or drugs, including rapamycin, curcumin, piperine, lithium (ongoing phase I clinical trial (NCT04273932), trehalose, corynoxine B, sodium valproate, and carbamazepine [10]. Studies in PD animal models and brain specimens from PD patients have revealed increased levels and activity of c-Abl in dopaminergic neurons with phosphorylation of protein substrates, such as α-synuclein and the E3 ubiquitin ligase [61]. The inhibition of c-Abl kinase activity by drugs used in the treatment of human leukaemia has shown promising neuroprotective effects in cell and animal models of PD [61]. This has led to the development of several ongoing phase 1 and phase 2 clinical trials testing the safety and efficacy of c-Abl inhibitors (i.e., imatinib, nilotinib, bafetinib, IkT-148009) in PD patients. In particular, the results from two recent trials have been recently published. The single-center, phase 2, double-blind trial NCT02954978 included 75 patients randomized vs. placebo and oral nilotinib 150-mg or nilotinib 300-mg for 12 months followed by a 3-month washout period [62]. This trial met its primary outcome (safety, tolerability, and detection in CSF), and is expected to guide a future phase 3 study to evaluate oral nilotinib as a disease-modifying medication for PD [62]. Nilotinib appeared to be reasonably safe and detectable in the cerebrospinal fluid, and exploratory biomarkers were altered in response to it [62]. Different results came from a 6-month, multicenter, double-blind trial (NCT03205488) that included 76 PD patients randomized to placebo vs. 150-mg nilotinib or 300-mg nilotinib once daily orally for 6 months, followed by a 2-month off-drug evaluation [63]. Unfortunately, nilotinib at 150 mg and 300 mg worsened on-medication PD motor scores (MDS-UPDRS-3) compared with placebo, with no differences in the change in off-medication MDS-UPDRS-3 scores [63]. Furthermore, there was no evidence of a treatment-related alteration of dopamine metabolites in the CSF. The authors concluded that, due to the low CSF exposure, lack of biomarker effect, and lack of efficacy, nilotinib should not be further tested in PD [63]. Furthermore, GCase may also decrease soluble α-synuclein in mice expressing mutant human A53T α-synuclein [64]. Two non-inhibitory GCase modulators (NCGC00188758 and NCGC607) have been found to increase GCase activity and decrease α-synuclein accumulation and toxicity in human neurons derived from induced pluripotent stem cells (iPSCs) [27]. 

Another different approach relies on the blockage of α-synuclein propagation between neurons. This approach is supported by preclinical studies, which showed that memantine, an antagonist of the NMDA (N-Methyl-D-Aspartate) subtype of glutamate receptor, may exert neuroprotective properties via the inhibition of cell-to-cell transmission of extracellular α-synuclein. A phase 3 trial (NCT03858270) is ongoing in order to evaluate the drug’s clinical impact on PD patients. A further promising approach to target α-synuclein aggregates is based on passive immunization using antibodies against α-synuclein, which may promote its lysosomal clearance. Antibodies against α-synuclein have already been tested in two recent RCTs, PRX002 (Roche) and BIIB054 (Biogen). Unfortunately, a randomized, double-blind, placebo-controlled study (SPARK) that evaluated the efficacy and safety of BIIB054 in PD patients did not meet both primary and secondary outcome measures for year one and also failed to meet secondary outcome measures [65]. This has led to the discontinuation of the development of BIIB054 for PD, and the SPARK study has been closed. Even the phase 2 PASADENA trial (NCT03100149), evaluating the safety and efficacy of intravenous prasinezumab (PRX002) in early-stage PD, did not show promising results [66]. This trial included 316 participants; 105 were assigned to receive placebo, 105 to receive 1500 mg of prasinezumab, and 106 to receive 4500 mg of prasinezumab [66]. Treatment with prasinezumab had no meaningful effect on global or imaging measures of PD progression compared to the placebo and was associated with infusion reactions [66]. However, another phase 2b, randomized, double-blind, placebo-controlled study with prasinezumab (NCT04777331), the PADOVA trial, is ongoing. As opposed to passive immunization, which involves administering anti-α-syn antibodies to the patient conferring temporary protection against the disease, active immunization involves stimulation of the immune system to produce antibodies against toxic α-syn conformations [67]. In this contest, different phase 1 clinical trials (PD03A [SYMPATH grant agreement 602999], PD01A [NCT01568099], UB-312 [NCT04075318]) targeted against oligomeric α-synuclein have been completed and have shown promising results with a positive antibody response [68,69]. Obviously, phase 2 trials are needed to test these promising results in a large cohort. Globally, due to the negative results of different anti-α-synuclein trials, a debate is ongoing as to whether toxic α-synuclein aggregation is the real culprit or rather if it is a loss of function in PD (gain-of-function vs. loss-of-function theories). The latter has been supported by the fact that the overexpression of native α-synuclein can rescue animal models of PD [70,71,72]. The loss-of-function α-synuclein theory is also based on the fact that α-synuclein is a critical protein in neuron (i.e., dopamine neurons) survival and that maintaining a certain level of biologically functional protein is an important consideration in targeting α-synuclein for therapies [70,71,72]. In this setting it has been assumed that a reduction in biologically functional α-synuclein, whether through aggregation or reduced expression, may at least in part be involved in the neurodegeneration of PD [70,71,72].

## 5. PRKN and PINK1 Genes

### 5.1. Pathophysiological Mechanisms

Up to 15% of monogenic PD cases are due to mutations in the PRKN gene [21,73]. From a clinical point of view, PD patients carrying biallelic mutations in the PARK gene show a typical early or juvenile age at onset (mean, 31 years), while tremor, bradykinesia and foot dystonia are the most commonly presenting signs [74]. Early dyskinesias, good response to dopaminergic treatment, and prominent motor fluctuations are other common findings in PARK gene mutation carriers [74]. On the contrary, cognitive alterations and dementia are very rare [74]. In addition, there is a debate on the role of monoallelic mutations in the PRKN gene; indeed, while some authors have suggested an association with an increased risk of PD [75,76]; other recent cohort studies have pointed out that heterozygous pathogenic PRKN mutations are common in the population but do not increase the risk of Parkinson’s disease [77,78]. PD due to PINK 1 biallelic mutations is characterized by a slow progression, good and persistent levodopa response, and minimal cognitive involvement [5]. In addition, some other features have been described, including dystonia, sleep benefit, and hyperreflexia [5]. 

The PRKN gene encodes for the E3 ubiquitin ligase parkin [79], which acts together with the PINK1 protein in a pathway of the mitochondrial quality control (MQC) system, which has neuroprotective effects [79]. The MQC has several functions, including the regulation of interconnected and dynamic networks of mitochondria through fusion and fission; government of mitochondrial morphology, regulation of ATP levels, and control of the constant and timely turnover of mitochondria [80]. The parkin protein, which is localized in the cytosol, is recruited to move to the outer mitochondrial membrane (OMM) of depolarized mitochondria [81] and promotes the ubiquitination of OMM proteins involved in upregulating mitochondrial fusion [82]. The ubiquitination is the step needed to remove these proteins and shift the balance between fission and fusion towards increased fission, promoting mitochondrial fragmentation and triggering the cellular autophagic machinery to mitophagy [83]. Parkin and PINK-1 may play similar roles in the cell, acting in a common pathway, where parkin acts downstream of PINK1. Indeed, missense mutants of parkin and PINK1 exhibited a loss of mitochondrial integrity because of reduced mitochondrial fission [82]. Parkin and PINK1 are involved in a direct interaction, whereby PINK1 phosphorylates parkin and activates its E3 ligase activity at the OMM [84]. If either parkin or PINK1 are mutated or down-regulated, the dysfunctional mitochondria will remain in the cytoplasm, creating an environment of oxidative stress ultimately resulting in cell death. *Parkin* has limited activity in the absence of PINK1, and this activity is potentiated through PINK1 expression [85]. 

In vivo PD PINK1 models using *Drosophila* display higher levels of misfolded mitochondrial respiratory complex components, ultimately leading to mitochondrial dysfunction and fragmentation [86]. PINK1 knockout (KO) mouse models showed a lower basal mitochondrial respiration in the dorsal striatum when compared to wild-type control mice [87,88]. The circuit also exhibited less dopamine release, suggesting that the neurotransmitter’s deficient release might be mostly caused by mitochondrial dysfunction and lower ATP levels [87]. Both PINK1 and parkin patient-derived midbrain dopaminergic neurons were found to have higher levels of α-synuclein, altered mitochondrial morphology, and increased vulnerability to mitochondrial aggressors [87]. Iron accumulation secondary to mitophagy dysfunction, both in PINK1- and PRKN-mutated PD patients, has raised the possibility to employ iron chelator drugs in this subgroup of PD patients [87,89].

### 5.2. Novel Therapeutic Approaches and Ongoing Clinical Trials

At the moment, no ongoing preclinical or clinical studies targeting PRKN or PINK1 mutations or the related biochemical pathways have been reported. However, there are some interesting pre-clinical studies that might lead to future clinical applications. It has already been pointed out that mitophagy and mitochondrial dysfunction are major aspects of PRKN and PINK1-related PD. Thus, restoring normal mitophagy through targeting the PINK1/Parkin pathway seems to be the most promising therapeutic approach [90,91]. The mitochondrial receptor Nip3-like protein X (Nix) overexpression was found to rescue the organelle function in fibroblast lines from homozygous mutated PINK1 patients [87]. Similarly, the matrix protein nipsnap homolog 1 (NIPSNAP1) might rescue PINK1-Parkin-dependent mitophagy in PINK1 phenotypes, since the molecule has been shown to contribute to this mitophagy pathway in cellular models [92]. Zebrafish lacking NIPSNAP1 were shown to have reduced brain mitophagy and increased reactive oxygen species (ROS) [92]. However, the removal of too many mitochondria may have a paradoxical negative effect if normal homeostasis is affected by the intervention [87]. In this setting, the identification of biomarkers for mitochondrial dysfunctions to serve as a therapeutic response monitor is of paramount importance [87]. 

## 6. DJ1 Gene 

### 6.1. Pathophysiological Mechanisms

Clinically, PD patients with biallelic DJ-1 mutations exhibit early-onset dyskinesia, rigidity, and tremor, followed by later manifestation of psychiatric symptoms, such as psychotic disturbance, anxiety, and cognitive decline, and generally respond well to L-DOPA treatment [93]. DJ-1 was originally identified as an oncogene and later associated with PD and diabetes mellitus [94]. The DJ-1 protein is expressed in reactive astrocytes and, to a lower extent, in neurons [95,96]. It is involved in mitochondrial function, apoptosis regulation, pro-survival signaling, autophagy, inflammatory responses, protection against oxidative stress, and chaperone activity [97]. PD-associated DJ-1 variants result in a loss of protein function. Experiments using PD-patient-derived DJ-1-deficient cells showed predominantly mitochondrial dysfunction and a reduced dopaminergic differentiation potential [97]. One study found elevated levels of α-synuclein in iPSC-derived human neurons from biallelic DJ-1 mutation carriers [98]. DJ-1 loss of function is also associated with increased inflammatory responses. siRNA DJ-1-knockdown mouse astrocytes are less able to protect against neurotoxins such as rotenone when compared to wild-type controls [96]. Furthermore, there is a reduced expression of prostaglandin D2 synthase, which regulates anti-inflammatory responses. Similarly, DJ-1-deficient microglia have an increased sensitivity to pro-inflammatory signals such as lipopolysaccharide (LPS), as well as an impaired uptake and degradation of α-synuclein and autophagy [99]. It has been demonstrated that rasagiline, a monoamine oxidase inhibitor, reduces the pro-inflammatory phenotype in microglia in a DJ-1 knockout model [100]. Therefore, this drug may be particularly useful in the treatment of DJ-1 PD patients [101].

### 6.2. Novel Therapeutic Approaches and Ongoing Clinical Trials

There are currently no human trials targeting DJ-1 for disease-modifying molecules. However, there are some interesting pre-clinical studies that might lead to future clinical applications. The most frequent approach in different pathological models is to increase DJ-1 levels in order to achieve neuroprotection in the face of oxidative stress [93]. Several studies using rat PD models have demonstrated the efficacy of recombinant wild-type DJ-1 for the protection of dopaminergic neurons [102,103,104]. However, all studies used intranigral injection for drug delivery, which is not clinically feasible [102,103,104]. A more promising way of DJ-1 delivery is based on the transactivator of transcription (TAT) cell-permeable peptide, used by HIV to cross plasma membranes [105]. Reduced dopaminergic dysfunction and improved behavior were achieved in a hemiparkinsonian mouse model, as well as reduced MPTP toxicity [106]. A second major strategy is the identification of drugs that inhibit the excessive oxidation of an important cysteine (Cys) residue at position 106 of the DJ-1 protein (Cys106). The reduced form of the protein (DJ-1 Cys106-SH) can be oxidized to a sulfinic acid form (DJ-1 Cys106-SO_2_H) and a sulfonic acid form (DJ-1 Cys106-SO_3_) in the presence of moderate or high oxidative stress (overoxidation) [93]. The reduced and sulfinic DJ-1 forms are stable; the sulfonic, on the other hand, is unstable and prone to aggregation (DJ-1 inactivation) [93]. The Cys106 sulfinate form (Cys-SO_2_^−^) stabilizes both human and *Drosophila* DJ-1 [107]. In this line of thought, studies using DJ-1 stabilizers in rat models were able to prevent dopaminergic neuronal death and even restore normal locomotor function. The most promising substance, compound-23, inhibited MPTP-induced locomotor deficits and cell death in the substantia nigra and striatum [93].

## 7. Conclusions

In this narrative review, we have illustrated the recent main advances in the treatment of genetic forms of PD. Each form has different pathophysiological characteristics that have not yet been fully elucidated. A precise identification of the pathophysiological mechanisms underlying specific genetic forms of PD represents a necessary effort in order to be able to develop customized gene-based treatments aimed at repairing different monogenic forms. While for the more frequent forms (i.e., GBA, LRRK2) experimental pharmacological trials are in progress or are about to begin, for the rarer forms (such as DJ1, PRKN, and PINK1), unfortunately, concrete therapeutic advances are still lacking.

## Figures and Tables

**Table 1:** Updated list of genes associated with PD. Reproduced with permission from Day JO and Mullin S, Genes (Basel); published by MDPI, 2021 [20].

	Gene (HGNC Approved Name)	Alternative Gene Names	Inheritance	Pathogenicity	PD Phenotype	Function
High penetrance	*SNCA*	*PARK1, PARK4, NCAP*	AD	Pathogenic	Early-onset	Uncertain (encodes α-synuclein)
	*VPS35*	*PARK17, MEM3*	AD	Pathogenic	Typical	Retromer and endosomal trafficking
	*PINK1*	*PARK6*	AR	Pathogenic	Early-onset	Mitochondrial
	*PARK7*	*DJ-1*	AR	Pathogenic	Early-onset	
	*PRKN*	*PARK2, PARKIN*	AR	Pathogenic	Early-onset	
	*PLA2G6*	*PARK14, IPLA2*	AR	Pathogenic	Early-onset, atypical	Cell membrane
	*ATP13A2*	*PARK9*	AR	Pathogenic	Early-onset, atypical	Lysosomal
	*FBX07*	*PARK15, FBX7*	AR	Pathogenic	Early-onset, atypical	Mitochondrial
	*POLG*	*POLG1, POLGA*	AD	Pathogenic	Early-onset, atypical	Mitochondrial DNA Maintenance
	*DNAJC6*	*PARK19, DJC6*	AR	Likely pathigenic	Early-onset	Synaptic vesicle formation and trafficking
	*DNAJC13*	*PARK21, RME8*	AD	Conflicting reports	Typical	
	*TMEM230*	*C20ORF30*	AD	Conflicting reports	Typical	
	*SYNJ1*	*PARK20*	AD	Conflicting reports	Typical	
	*VPS13C*	*PARK23*	AR	Pathogenic	Early-onset	Mitochondrial
	*CHCHD2*	*-*	AD	Pathogenic	Typical	Uncertain
	*DCTN1*	*-*	AD	Pathogenic	Atypical	Microtubule
Variable penetrance	*LRRK2*	*PARK8, DARDARIN*	AD	Pathogenic	Typical	Lysosomal, mitochondrial, microtubule
	*GBA*	*GBA1*	AD	Pathogenic	Typical	Lysosomal
Associated with PD but unlikely to be pathogenic	*HTRA2*	*-*	AD	Uncertain/likely benign	-	Mitochondrial
	*UCHL1*	*PARK5*	AD	Uncertain/likely benign	-	Ubiquitin-proteasome
	*GIGYF2*	*PARK11*	AD	Uncertain/likely benign	-	Uncertain
	*EIF4G1*	*-*	AD	Benign	-	mRNA translation
	*LRP10*	*LRP9*	AD ^1^	Uncertain	-	Uncertain

AD ^1^ = autosomal dominant, AR = autosomal recessive, HNGC = HUGO Gene Nomenclature Committee.

**Table 2:** Clinical trials of therapeutic interventions in GBA-PD patients.

Therapeutic Agent	Target/Mode of Action	Clinical Trial ID	Stage	Status
Ambroxol Hydrochloride	GCase enhancement: increase GCase activity and reduce α-synuclein levels in vitro and in vivo	AMBITIOUS NCT05287503	2	Recruiting
Ambroxol Hydrochloride	GCase enhancement: increase GCase activity and reduce α-synuclein levels in vitro and in vivo	NCT02914366	2	Active, not recruiting
Intracisternal AAV9-GBA1 gene (LY3884961) administration	β-Glucocerebrosidase gene therapy	PROPEL NCT04127578	1/2a	Active, not recruiting
Venglustat GZ/SAR402671	Glucosylceramide synthase inhibition: allosteric inhibitor of the enzyme glucosylceramide synthase	MOVES-PD NCT02906020	2	Terminated (The topline results of the 52-week double-blind placebo-controlled period were analyzed. The study did not meet the primary or secondary endpoints. Based on these results, the decision was made to halt the long-term follow-up period of the study)

**Table 3:** Clinical trials of therapeutic interventions in LRRK2-PD patients.

Therapeutic Agent	Target/Mode of Action	Clinical Trial ID	Stage	Status
BIIB122/DNL151	Inhibition of LRRK2 kinase	NCT05348785	2b	Recruiting
BIIB122/DNL151	Inhibition of LRRK2 kinase	NCT05418673	3	Recruiting
Trehalose	Autophagy enhancement	NCT05355064	4	Not yet recruiting
BIIB122/DNL151	Inhibition of LRRK2 kinase; autophagy promoter	NCT04056689	1b	Completed
DNL201	Inhibition of LRRK2 kinase; autophagy promoter	NCT03710707	1b	Completed
BIIB094	Antisense oligonucleotide for LRRK2 inhibition	NCT03976349	1	Recruiting

**Table 4:** Clinical trials of therapeutic interventions against a-synuclein.

Therapeutic Agent	Target/Mode of Action	Clinical Trial ID	Stage	Status
Vodobatinib	Inhibition of α-synuclein aggregation (autophagy-ABL1 inhibitors)	NCT03655236	2	Recruiting
Radotinib	Inhibition of α-synuclein aggregation (autophagy-ABL1 inhibitors)	NCT04691661	2	Recruiting
Nilotinib	Inhibition of α-synuclein aggregation (autophagy-ABL1 inhibitors)	NCT02954978	2	Met primary outcome
Nilotinib	Inhibition of α-synuclein aggregation (autophagy-ABL1 inhibitors)	NCT03205488	2	Failed meeting primary endpoint
IkT-148009	Inhibition of α-synuclein aggregation (autophagy-ABL1 inhibitors)	NCT04350177	1	Active, not recruiting
Lithium	Enhancement autophagy and reduced intracellular levels of a-synuclein	NCT04273932	1	Active, not recruiting
Memantine	Inhibition of α-synuclein cell–cell Transmission	NCT03858270	3	Recruiting
BIIB054 (cinpanemab)	Monoclonal antibody that binds to α-synuclein	NCT03318523	2	Failed meeting primary endpoint
Prasinezumab (RO7046015/PRX002)	Monoclonal antibody that binds to α-synuclein	NCT03100149	2	Failed meeting primary endpoint
Prasinezumab (PADOVA TRIAL)	Monoclonal antibody that binds to α-synuclein	NCT04777331	2	Recruiting
PD01A	Active α-synuclein immunization	NCT01568099	1	Positive antibody response
PD03A	Active α-synuclein immunization	SYMPATH grant agreement 602999	1	Positive antibody response
UB-312	Active α-synuclein immunization	NCT04075318	1	Positive antibody response
Anle138b	Structure-dependent binding to pathological aggregates and strong inhibition of formation of pathological oligomers in vitro and in vivo for α-synuclein	NCT04208152	1	Completed
NPT200-11	α -synuclein misfolding inhibition	NPT200-11	1	Completed
UCB0599	α -synuclein misfolding inhibition	NCT04875962	1	Completed
UCB0599 (ORCHESTRA STUDY)	α -synuclein misfolding inhibition	NCT04658186	2	Recruiting
K0706	Inhibition of α-synuclein aggregation (autophagy-ABL1 inhibitors)	NCT03655236	2	Recruiting
IkT-148009	Inhibition of α-synuclein aggregation (autophagy-ABL1 inhibitors)	NCT04350177	1	Active, not recruiting
AFFITOPE^®^ PD01A	New vaccine against α -synuclein	NCT02758730	1	Withdrawn

## Data Availability

Not applicable.

## References

[1] Kalia L.V., Lang A.E. (2015). Parkinson’s Disease. Lancet.

[2] Cacabelos R. (2017). Parkinson’s Disease: From Pathogenesis to Pharmacogenomics. Int. J. Mol. Sci..

[3] Hustad E., Aasly J.O. (2020). Clinical and Imaging Markers of Prodromal Parkinson’s Disease. Front. Neurol..

[4] Horsager J., Andersen K.B., Knudsen K., Skjærbæk C., Fedorova T.D., Okkels N., Schaeffer E., Bonkat S.K., Geday J., Otto M. (2020). Brain-First versus Body-First Parkinson’s Disease: A Multimodal Imaging Case-Control Study. Brain.

[5] Kim C.Y., Alcalay R.N. (2017). Genetic Forms of Parkinson’s Disease. Semin. Neurol..

[6] Angelopoulou E., Paudel Y.N., Papageorgiou S.G., Piperi C. (2022). Environmental Impact on the Epigenetic Mechanisms Underlying Parkinson’s Disease Pathogenesis: A Narrative Review. Brain Sci..

[7] Kochmanski J., Kuhn N.C., Bernstein A.I. (2022). Parkinson’s Disease-Associated, Sex-Specific Changes in DNA Methylation at PARK7 (DJ-1), SLC17A6 (VGLUT2), PTPRN2 (IA-2β), and NR4A2 (NURR1) in Cortical Neurons. NPJ Parkinsons. Dis..

[8] Miranda-Morales E., Meier K., Sandoval-Carrillo A., Salas-Pacheco J., Vázquez-Cárdenas P., Arias-Carrión O. (2017). Implications of DNA Methylation in Parkinson’s Disease. Front. Mol. Neurosci..

[9] Le Heron C., MacAskill M., Mason D., Dalrymple-Alford J., Anderson T., Pitcher T., Myall D. (2021). A Multi-Step Model of Parkinson’s Disease Pathogenesis. Mov. Disord..

[10] Gouda N.A., Elkamhawy A., Cho J. (2022). Emerging Therapeutic Strategies for Parkinson’s Disease and Future Prospects: A 2021 Update. Biomedicines.

[11] Wong Y.C., Krainc D. (2017). α-Synuclein Toxicity in Neurodegeneration: Mechanism and Therapeutic Strategies. Nat. Med..

[12] Polymeropoulos M.H., Lavedan C., Leroy E., Ide S.E., Dehejia A., Dutra A., Pike B., Root H., Rubenstein J., Boyer R. (1997). Mutation in the Alpha-Synuclein Gene Identified in Families with Parkinson’s Disease. Science.

[13] Sulzer D., Edwards R.H. (2019). The Physiological Role of A-synuclein and Its Relationship to Parkinson’s Disease. J. Neurochem..

[14] Cannon J.R., Greenamyre J.T. (2013). Gene–Environment Interactions in Parkinson’s Disease: Specific Evidence in Humans and Mammalian Models. Neurobiol. Dis..

[15] Schirinzi T., Martella G., Pisani A. (2016). Double Hit Mouse Model of Parkinson’s Disease. Oncotarget.

[16] Cavallieri F., Fioravanti V., Toschi G., Grisanti S., Napoli M., Moratti C., Pascarella R., Versari A., Fraternali A., Casali M. (2022). COVID-19 and Parkinson’s Disease: A Casual Association or a Possible Second Hit in Neurodegeneration?. J. Neurol..

[17] Armstrong M.J., Okun M.S. (2020). Diagnosis and Treatment of Parkinson Disease: A Review. JAMA.

[18] Yoon S.Y., Suh J.H., Yang S.N., Han K., Kim Y.W. (2021). Association of Physical Activity, Including Amount and Maintenance, With All-Cause Mortality in Parkinson Disease. JAMA Neurol..

[19] Oliveira de Carvalho A., Filho A.S.S., Murillo-Rodriguez E., Rocha N.B., Carta M.G., Machado S. (2018). Physical Exercise For Parkinson’s Disease: Clinical And Experimental Evidence. Clin. Pract. Epidemiol. Ment. Health.

[20] Day J.O., Mullin S. (2021). The Genetics of Parkinson’s Disease and Implications for Clinical Practice. Genes.

[21] Blauwendraat C., Nalls M.A., Singleton A.B. (2020). The Genetic Architecture of Parkinson’s Disease. Lancet Neurol..

[22] Cherian A., Divya K.P. (2020). Genetics of Parkinson’s Disease. Acta Neurol. Belg..

[23] Billingsley K.J., Ding J., Alvarez Jerez P., Illarionova A., Levine K., Grenn F.P., Makarious M.B., Moore A., Vitale D., Reed X. (2023). Genome-wide Analysis of Structural Variants in Parkinson’s Disease. Ann. Neurol..

[24] Vollstedt E.-J., Schaake S., Lohmann K., Padmanabhan S., Brice A., Lesage S., Tesson C., Vidailhet M., Wurster I., Hentati F. (2023). Embracing Monogenic Parkinson’s Disease: The MJFF Global Genetic PD Cohort. Mov. Disord..

[25] Riboldi G.M., Di Fonzo A.B. (2019). GBA, Gaucher Disease, and Parkinson’s Disease: From Genetic to Clinic to New Therapeutic Approaches. Cells.

[26] Neudorfer O., Giladi N., Elstein D., Abrahamov A., Turezkite T., Aghai E., Reches A., Bembi B., Zimran A. (1996). Occurrence of Parkinson’s Syndrome in Type I Gaucher Disease. QJM Int. J. Med..

[27] Gegg M.E., Menozzi E., Schapira A.H.V. (2022). Glucocerebrosidase-Associated Parkinson Disease: Pathogenic Mechanisms and Potential Drug Treatments. Neurobiol. Dis..

[28] Menozzi E., Schapira A.H.V. (2021). Exploring the Genotype-Phenotype Correlation in GBA-Parkinson Disease: Clinical Aspects, Biomarkers, and Potential Modifiers. Front. Neurol..

[29] Cilia R., Tunesi S., Marotta G., Cereda E., Siri C., Tesei S., Zecchinelli A.L., Canesi M., Mariani C.B., Meucci N. (2016). Survival and Dementia in GBA-Associated Parkinson’s Disease: The Mutation Matters. Ann. Neurol..

[30] Senkevich K., Gan-Or Z. (2020). Autophagy Lysosomal Pathway Dysfunction in Parkinson’s Disease; Evidence from Human Genetics. Park. Relat. Disord..

[31] Petrucci S., Ginevrino M., Trezzi I., Monfrini E., Ricciardi L., Albanese A., Avenali M., Barone P., Bentivoglio A.R., Bonifati V. (2020). GBA-Related Parkinson’s Disease: Dissection of Genotype-Phenotype Correlates in a Large Italian Cohort. Mov. Disord..

[32] McNeill A., Magalhaes J., Shen C., Chau K.-Y., Hughes D., Mehta A., Foltynie T., Cooper J.M., Abramov A.Y., Gegg M. (2014). Ambroxol Improves Lysosomal Biochemistry in Glucocerebrosidase Mutation-Linked Parkinson Disease Cells. Brain.

[33] Peterschmitt M.J., Saiki H., Hatano T., Gasser T., Isaacson S.H., Gaemers S.J.M., Minini P., Saubadu S., Sharma J., Walbillic S. (2022). Safety, Pharmacokinetics, and Pharmacodynamics of Oral Venglustat in Patients with Parkinson’s Disease and a GBA Mutation: Results from Part 1 of the Randomized, Double-Blinded, Placebo-Controlled MOVES-PD Trial. J. Park. Dis..

[34] Gegg M.E., Burke D., Heales S.J.R., Cooper J.M., Hardy J., Wood N.W., Schapira A.H.V. (2012). Glucocerebrosidase Deficiency in Substantia Nigra of Parkinson Disease Brains. Ann. Neurol..

[35] Sanchez-Martinez A., Beavan M., Gegg M.E., Chau K.-Y., Whitworth A.J., Schapira A.H.V. (2016). Parkinson Disease-Linked GBA Mutation Effects Reversed by Molecular Chaperones in Human Cell and Fly Models. Sci. Rep..

[36] Gegg M.E., Schapira A.H.V. (2018). The Role of Glucocerebrosidase in Parkinson Disease Pathogenesis. FEBS J..

[37] Mullin S., Smith L., Lee K., D’Souza G., Woodgate P., Elflein J., Hällqvist J., Toffoli M., Streeter A., Hosking J. (2020). Ambroxol for the Treatment of Patients With Parkinson Disease With and Without Glucocerebrosidase Gene Mutations: A Nonrandomized, Noncontrolled Trial. JAMA Neurol..

[38] Mullin S., Stokholm M.G., Hughes D., Mehta A., Parbo P., Hinz R., Pavese N., Brooks D.J., Schapira A.H.V. (2021). Brain Microglial Activation Increased in Glucocerebrosidase (GBA) Mutation Carriers without Parkinson’s Disease. Mov. Disord..

[39] Abeliovich A., Hefti F., Sevigny J. (2021). Gene Therapy for Parkinson’s Disease Associated with GBA1 Mutations. J. Park. Dis..

[40] Jackson K.L., Viel C., Clarke J., Bu J., Chan M., Wang B., Shihabuddin L.S., Sardi S.P. (2019). Viral Delivery of a MicroRNA to Gba to the Mouse Central Nervous System Models Neuronopathic Gaucher Disease. Neurobiol. Dis..

[41] Rockenstein E., Clarke J., Viel C., Panarello N., Treleaven C.M., Kim C., Spencer B., Adame A., Park H., Dodge J.C. (2016). Glucocerebrosidase Modulates Cognitive and Motor Activities in Murine Models of Parkinson’s Disease. Hum. Mol. Genet..

[42] Rocha E.M., Smith G.A., Park E., Cao H., Brown E., Hayes M.A., Beagan J., McLean J.R., Izen S.C., Perez-Torres E. (2015). Glucocerebrosidase Gene Therapy Prevents α-Synucleinopathy of Midbrain Dopamine Neurons. Neurobiol. Dis..

[43] Jeong G.R., Lee B.D. (2020). Pathological Functions of LRRK2 in Parkinson’s Disease. Cells.

[44] Lee C.-Y., Menozzi E., Chau K.-Y., Schapira A.H.V. (2021). Glucocerebrosidase 1 and Leucine-Rich Repeat Kinase 2 in Parkinson Disease and Interplay between the Two Genes. J. Neurochem..

[45] Rui Q., Ni H., Li D., Gao R., Chen G. (2018). The Role of LRRK2 in Neurodegeneration of Parkinson Disease. Curr. Neuropharmacol..

[46] Connor-Robson N., Booth H., Martin J.G., Gao B., Li K., Doig N., Vowles J., Browne C., Klinger L., Juhasz P. (2019). An Integrated Transcriptomics and Proteomics Analysis Reveals Functional Endocytic Dysregulation Caused by Mutations in LRRK2. Neurobiol. Dis..

[47] Ravinther A.I., Dewadas H.D., Tong S.R., Foo C.N., Lin Y.-E., Chien C.-T., Lim Y.M. (2022). Molecular Pathways Involved in LRRK2-Linked Parkinson’s Disease: A Systematic Review. Int. J. Mol. Sci..

[48] Erb M.L., Moore D.J. (2020). LRRK2 and the Endolysosomal System in Parkinson’s Disease. J. Park. Dis..

[49] Jennings D., Huntwork-Rodriguez S., Henry A.G., Sasaki J.C., Meisner R., Diaz D., Solanoy H., Wang X., Negrou E., Bondar V.V. (2022). Preclinical and Clinical Evaluation of the LRRK2 Inhibitor DNL201 for Parkinson’s Disease. Sci. Transl. Med..

[50] Zhao H.T., John N., Delic V., Ikeda-Lee K., Kim A., Weihofen A., Swayze E.E., Kordasiewicz H.B., West A.B., Volpicelli-Daley L.A. (2017). LRRK2 Antisense Oligonucleotides Ameliorate α-Synuclein Inclusion Formation in a Parkinson’s Disease Mouse Model. Mol. Ther. Nucleic Acids.

[51] Magistrelli L., Contaldi E., Comi C. (2021). The Impact of SNCA Variations and Its Product Alpha-Synuclein on Non-Motor Features of Parkinson’s Disease. Life.

[52] Guadagnolo D., Piane M., Torrisi M.R., Pizzuti A., Petrucci S. (2021). Genotype-Phenotype Correlations in Monogenic Parkinson Disease: A Review on Clinical and Molecular Findings. Front. Neurol..

[53] Poewe W., Seppi K., Tanner C.M., Halliday G.M., Brundin P., Volkmann J., Schrag A.-E., Lang A.E. (2017). Parkinson Disease. Nat. Rev. Dis. Primers.

[54] Bridi J.C., Hirth F. (2018). Mechanisms of α-Synuclein Induced Synaptopathy in Parkinson’s Disease. Front. Neurosci..

[55] Wilkaniec A., Lenkiewicz A.M., Babiec L., Murawska E., Jęśko H.M., Cieślik M., Culmsee C., Adamczyk A. (2021). Exogenous Alpha-Synuclein Evoked Parkin Downregulation Promotes Mitochondrial Dysfunction in Neuronal Cells. Implications for Parkinson’s Disease Pathology. Front. Aging Neurosci..

[56] Chinta S.J., Mallajosyula J.K., Rane A., Andersen J.K. (2010). Mitochondrial Alpha-Synuclein Accumulation Impairs Complex I Function in Dopaminergic Neurons and Results in Increased Mitophagy in Vivo. Neurosci. Lett..

[57] Choubey V., Safiulina D., Vaarmann A., Cagalinec M., Wareski P., Kuum M., Zharkovsky A., Kaasik A. (2011). Mutant A53T α-Synuclein Induces Neuronal Death by Increasing Mitochondrial Autophagy. J. Biol. Chem..

[58] Uehara T., Choong C.-J., Nakamori M., Hayakawa H., Nishiyama K., Kasahara Y., Baba K., Nagata T., Yokota T., Tsuda H. (2019). Amido-Bridged Nucleic Acid (AmNA)-Modified Antisense Oligonucleotides Targeting α-Synuclein as a Novel Therapy for Parkinson’s Disease. Sci. Rep..

[59] Uehara T., Choong C.J., Hayakawa H., Kasahara Y., Nagata T., Yokota T., Baba K., Nakamori M., Obika S., Mochizuki H. (2017). Antisense Oligonucleotides Containing Amido-Bridged Nucleic Acid Reduce SNCA Expression and Improve Motor Function in Parkinson’s Disease Animal Models. J. Neurol. Sci..

[60] Arotcarena M.-L., Bourdenx M., Dutheil N., Thiolat M.-L., Doudnikoff E., Dovero S., Ballabio A., Fernagut P.-O., Meissner W.G., Bezard E. (2019). Transcription Factor EB Overexpression Prevents Neurodegeneration in Experimental Synucleinopathies. JCI Insight.

[61] Lindholm D., Pham D.D., Cascone A., Eriksson O., Wennerberg K., Saarma M. (2016). C-Abl Inhibitors Enable Insights into the Pathophysiology and Neuroprotection in Parkinson’s Disease. Front. Aging Neurosci..

[62] Pagan F.L., Hebron M.L., Wilmarth B., Torres-Yaghi Y., Lawler A., Mundel E.E., Yusuf N., Starr N.J., Anjum M., Arellano J. (2020). Nilotinib Effects on Safety, Tolerability, and Potential Biomarkers in Parkinson Disease: A Phase 2 Randomized Clinical Trial. JAMA Neurol..

[63] Simuni T., Fiske B., Merchant K., Coffey C.S., Klingner E., Caspell-Garcia C., Lafontant D.-E., Matthews H., Wyse R.K., Brundin P. (2021). Efficacy of Nilotinib in Patients With Moderately Advanced Parkinson Disease: A Randomized Clinical Trial. JAMA Neurol..

[64] Papadopoulos V.E., Nikolopoulou G., Antoniadou I., Karachaliou A., Arianoglou G., Emmanouilidou E., Sardi S.P., Stefanis L., Vekrellis K. (2018). Modulation of β-Glucocerebrosidase Increases α-Synuclein Secretion and Exosome Release in Mouse Models of Parkinson’s Disease. Hum. Mol. Genet..

[65] Lang A.E., Siderowf A.D., Macklin E.A., Poewe W., Brooks D.J., Fernandez H.H., Rascol O., Giladi N., Stocchi F., Tanner C.M. (2022). Trial of Cinpanemab in Early Parkinson’s Disease. N. Engl. J. Med..

[66] Pagano G., Taylor K.I., Anzures-Cabrera J., Marchesi M., Simuni T., Marek K., Postuma R.B., Pavese N., Stocchi F., Azulay J.-P. (2022). Trial of Prasinezumab in Early-Stage Parkinson’s Disease. N. Engl. J. Med..

[67] Mandler M., Valera E., Rockenstein E., Mante M., Weninger H., Patrick C., Adame A., Schmidhuber S., Santic R., Schneeberger A. (2015). Active Immunization against Alpha-Synuclein Ameliorates the Degenerative Pathology and Prevents Demyelination in a Model of Multiple System Atrophy. Mol. Neurodegener..

[68] Meissner W.G., Traon A.P.-L., Foubert-Samier A., Galabova G., Galitzky M., Kutzelnigg A., Laurens B., Lührs P., Medori R., Péran P. (2020). A Phase 1 Randomized Trial of Specific Active α-Synuclein Immunotherapies PD01A and PD03A in Multiple System Atrophy. Mov. Disord..

[69] Yu H.J., Thijssen E., van Brummelen E., van der Plas J.L., Radanovic I., Moerland M., Hsieh E., Groeneveld G.J., Dodart J. (2022). A Randomized First-in-Human Study With UB-312, a UBITh^®^ A-Synuclein Peptide Vaccine. Mov. Disord..

[70] Collier T.J., Redmond D.E., Steece-Collier K., Lipton J.W., Manfredsson F.P. (2016). Is Alpha-Synuclein Loss-of-Function a Contributor to Parkinsonian Pathology? Evidence from Non-Human Primates. Front. Neurosci..

[71] Kanaan N.M., Manfredsson F.P. (2012). Loss of Functional Alpha-Synuclein: A Toxic Event in Parkinson’s Disease?. J. Park. Dis..

[72] Gorbatyuk O.S., Li S., Nash K., Gorbatyuk M., Lewin A.S., Sullivan L.F., Mandel R.J., Chen W., Meyers C., Manfredsson F.P. (2010). In Vivo RNAi-Mediated α-Synuclein Silencing Induces Nigrostriatal Degeneration. Mol. Ther..

[73] Deng H., Wang P., Jankovic J. (2018). The Genetics of Parkinson Disease. Ageing Res. Rev..

[74] Wasner K., Grünewald A., Klein C. (2020). Parkin-Linked Parkinson’s Disease: From Clinical Insights to Pathogenic Mechanisms and Novel Therapeutic Approaches. Neurosci. Res..

[75] Klein C., Lohmann-Hedrich K., Rogaeva E., Schlossmacher M.G., Lang A.E. (2007). Deciphering the Role of Heterozygous Mutations in Genes Associated with Parkinsonism. Lancet Neurol..

[76] Lubbe S.J., Bustos B.I., Hu J., Krainc D., Joseph T., Hehir J., Tan M., Zhang W., Escott-Price V., Williams N.M. (2021). Assessing the Relationship between Monoallelic *PRKN* Mutations and Parkinson’s Risk. Hum. Mol. Genet..

[77] Yu E., Rudakou U., Krohn L., Mufti K., Ruskey J.A., Asayesh F., Estiar M.A., Spiegelman D., Surface M., Fahn S. (2021). Analysis of Heterozygous *PRKN* Variants and Copy-Number Variations in Parkinson’s Disease. Mov. Disord..

[78] Zhu W., Huang X., Yoon E., Bandres-Ciga S., Blauwendraat C., Billingsley K.J., Cade J.H., Wu B.P., Williams V.H., Schindler A.B. (2022). Heterozygous *PRKN* Mutations Are Common but Do Not Increase the Risk of Parkinson’s Disease. Brain.

[79] Seirafi M., Kozlov G., Gehring K. (2015). Parkin Structure and Function. FEBS J..

[80] Chauhan A., Vera J., Wolkenhauer O. (2014). The Systems Biology of Mitochondrial Fission and Fusion and Implications for Disease and Aging. Biogerontology.

[81] Büeler H. (2009). Impaired Mitochondrial Dynamics and Function in the Pathogenesis of Parkinson’s Disease. Exp. Neurol..

[82] Poole A.C., Thomas R.E., Yu S., Vincow E.S., Pallanck L. (2010). The Mitochondrial Fusion-Promoting Factor Mitofusin Is a Substrate of the PINK1/Parkin Pathway. PLoS ONE.

[83] Kuang E., Qi J., Ronai Z. (2013). Emerging Roles of E3 Ubiquitin Ligases in Autophagy. Trends Biochem Sci.

[84] Sha D., Chin L.-S., Li L. (2010). Phosphorylation of Parkin by Parkinson Disease-Linked Kinase PINK1 Activates Parkin E3 Ligase Function and NF-KappaB Signaling. Hum. Mol. Genet..

[85] Xiong H., Wang D., Chen L., Choo Y.S., Ma H., Tang C., Xia K., Jiang W., Ronai Z., Zhuang X. (2009). Parkin, PINK1, and DJ-1 Form a Ubiquitin E3 Ligase Complex Promoting Unfolded Protein Degradation. J. Clin. Investig..

[86] Pimenta de Castro I., Costa A.C., Lam D., Tufi R., Fedele V., Moisoi N., Dinsdale D., Deas E., Loh S.H.Y., Martins L.M. (2012). Genetic Analysis of Mitochondrial Protein Misfolding in Drosophila Melanogaster. Cell Death Differ..

[87] Vizziello M., Borellini L., Franco G., Ardolino G. (2021). Disruption of Mitochondrial Homeostasis: The Role of PINK1 in Parkinson’s Disease. Cells.

[88] Zhi L., Qin Q., Muqeem T., Seifert E.L., Liu W., Zheng S., Li C., Zhang H. (2019). Loss of PINK1 Causes Age-Dependent Decrease of Dopamine Release and Mitochondrial Dysfunction. Neurobiol. Aging.

[89] Key J., Sen N.E., Arsović A., Krämer S., Hülse R., Khan N.N., Meierhofer D., Gispert S., Koepf G., Auburger G. (2020). Systematic Surveys of Iron Homeostasis Mechanisms Reveal Ferritin Superfamily and Nucleotide Surveillance Regulation to Be Modified by PINK1 Absence. Cells.

[90] Narendra D.P., Youle R.J. (2011). Targeting Mitochondrial Dysfunction: Role for PINK1 and Parkin in Mitochondrial Quality Control. Antioxid. Redox Signal..

[91] McLelland G.-L., Soubannier V., Chen C.X., McBride H.M., Fon E.A. (2014). Parkin and PINK1 Function in a Vesicular Trafficking Pathway Regulating Mitochondrial Quality Control. EMBO J..

[92] Abudu Y.P., Pankiv S., Mathai B.J., Lamark T., Johansen T., Simonsen A. (2019). NIPSNAP1 and NIPSNAP2 Act as “Eat Me” Signals to Allow Sustained Recruitment of Autophagy Receptors during Mitophagy. Autophagy.

[93] Repici M., Giorgini F. (2019). DJ-1 in Parkinson’s Disease: Clinical Insights and Therapeutic Perspectives. J. Clin. Med..

[94] Zhang L., Wang J., Wang J., Yang B., He Q., Weng Q. (2020). Role of DJ-1 in Immune and Inflammatory Diseases. Front. Immunol..

[95] Mullett S.J., Di Maio R., Greenamyre J.T., Hinkle D.A. (2013). DJ-1 Expression Modulates Astrocyte-Mediated Protection Against Neuronal Oxidative Stress. J. Mol. Neurosci..

[96] Mullett S.J., Hinkle D.A. (2011). DJ-1 Deficiency in Astrocytes Selectively Enhances Mitochondrial Complex I Inhibitor-Induced Neurotoxicity. J. Neurochem..

[97] Mencke P., Boussaad I., Romano C.D., Kitami T., Linster C.L., Krüger R. (2021). The Role of DJ-1 in Cellular Metabolism and Pathophysiological Implications for Parkinson’s Disease. Cells.

[98] Burbulla L.F., Song P., Mazzulli J.R., Zampese E., Wong Y.C., Jeon S., Santos D.P., Blanz J., Obermaier C.D., Strojny C. (2017). Dopamine Oxidation Mediates Mitochondrial and Lysosomal Dysfunction in Parkinson’s Disease. Science.

[99] Wang C., Yang T., Liang M., Xie J., Song N. (2021). Astrocyte Dysfunction in Parkinson’s Disease: From the Perspectives of Transmitted α-Synuclein and Genetic Modulation. Transl. Neurodegener..

[100] Trudler D., Weinreb O., Mandel S.A., Youdim M.B.H., Frenkel D. (2014). DJ-1 Deficiency Triggers Microglia Sensitivity to Dopamine toward a pro-Inflammatory Phenotype That Is Attenuated by Rasagiline. J. Neurochem..

[101] MacMahon Copas A.N., McComish S.F., Fletcher J.M., Caldwell M.A. (2021). The Pathogenesis of Parkinson’s Disease: A Complex Interplay Between Astrocytes, Microglia, and T Lymphocytes?. Front. Neurol..

[102] Inden M., Taira T., Kitamura Y., Yanagida T., Tsuchiya D., Takata K., Yanagisawa D., Nishimura K., Taniguchi T., Kiso Y. (2006). PARK7 DJ-1 Protects against Degeneration of Nigral Dopaminergic Neurons in Parkinson’s Disease Rat Model. Neurobiol. Dis..

[103] Gao H., Yang W., Qi Z., Lu L., Duan C., Zhao C., Yang H. (2012). DJ-1 Protects Dopaminergic Neurons against Rotenone-Induced Apoptosis by Enhancing ERK-Dependent Mitophagy. J. Mol. Biol..

[104] Sun S.-Y., An C.-N., Pu X.-P. (2012). DJ-1 Protein Protects Dopaminergic Neurons against 6-OHDA/MG-132-Induced Neurotoxicity in Rats. Brain Res. Bull..

[105] Antoniou X., Borsello T. (2010). Cell Permeable Peptides: A Promising Tool to Deliver Neuroprotective Agents in the Brain. Pharmaceuticals.

[106] Lev N., Barhum Y., Ben-Zur T., Aharony I., Trifonov L., Regev N., Melamed E., Gruzman A., Offen D. (2015). A DJ-1 Based Peptide Attenuates Dopaminergic Degeneration in Mice Models of Parkinson’s Disease via Enhancing Nrf2. PLoS ONE.

[107] Lin J., Prahlad J., Wilson M.A. (2012). Conservation of Oxidative Protein Stabilization in an Insect Homologue of Parkinsonism-Associated Protein DJ-1. Biochemistry.

